# Ribosomal RNA expansion segments and their role in ribosome biology

**DOI:** 10.1042/BST20231106

**Published:** 2024-05-02

**Authors:** Robert Rauscher, Norbert Polacek

**Affiliations:** Department for Chemistry, Biochemistry and Pharmaceutical Sciences, University of Bern, Freiestrasse 3, 3012 Bern, Switzerland

**Keywords:** cotranslational factor recruitment, nascent peptide chain maturation, rRNA expansion segments, translation elongation, translation regulation

## Abstract

Ribosomes are universally conserved cellular machines that catalyze protein biosynthesis. The active sites underly immense evolutionary conservation resulting in virtually identical core structures of ribosomes in all domains of life including organellar ribosomes. However, more peripheral structures of cytosolic ribosomes changed during evolution accommodating new functions and regulatory options. The expansion occurred at the riboprotein level, including more and larger ribosomal proteins and at the RNA level increasing the length of ribosomal RNA. Expansions within the ribosomal RNA occur as clusters at conserved sites that face toward the periphery of the cytosolic ribosome. Recent biochemical and structural work has shed light on how rRNA-specific expansion segments (ESs) recruit factors during translation and how they modulate translation dynamics in the cytosol. Here we focus on recent work on yeast, human and trypanosomal cytosolic ribosomes that explores the role of two specific rRNA ESs within the small and large subunit respectively. While no single regulatory strategy exists, the absence of ESs has consequences for proteomic stability and cellular fitness, rendering them fascinating evolutionary tools for tailored protein biosynthesis.

## rRNA expansion from bacteria to mammals

Ribosomes are remnants of the RNA world, and as such their catalytic center remained exclusively composed of RNA during evolution. The ability to perform peptide bond formation further seems to rely on a very defined structure and sequence within the peptidyl transferase center (PTC), one of the regions of highest conservation among species. *In vitro* evolution of RNAs that could perform peptide bond formation yielded similar sequence and structural contexts as observed in contemporary cytosolic ribosomes suggesting that the PTC is one of very few possible solutions to efficient RNA-mediated peptide bond formation [1].

The selection of cognate tRNAs within the decoding center is the second major activity performed by ribosomes. This region is highly conserved among species of all kingdoms of life. Research on the minimum requirements for nucleotide selection suggests that a stretch of three nucleotides (nt) pairing to a complementary substrate strand provides sufficient stability to accelerate the sequence-specific addition of amino acids to a growing peptide chain [2]. This 3-nt recognition is still found in contemporary protein biosynthesis and is referred to as codon-anticodon pairing between the mRNA and a tRNA that delivers amino acids. To be recognized as a cognate pairing, the first 2 nt of a codon rely on Watson–Crick base pairing whereas the third position has more freedom allowing for wobble base pairing with the first nucleotide of the anticodon. These functionalities were already present in ribosomes of the last universal common ancestor and are invariably found in contemporary ribosomes. The PTC and the decoding center are located within two distinct subunits; the large subunit (LSU) harbors the PTC and the small subunit (SSU) contains the decoding center. Each subunit is formed by ribosomal RNA and a set of proteins. While this common core architecture remained, cytosolic ribosomes of complex organisms differ tremendously from those found in single-cellular prokaryotes or organelles of eukaryotes [3,4].

**Figure 1.**
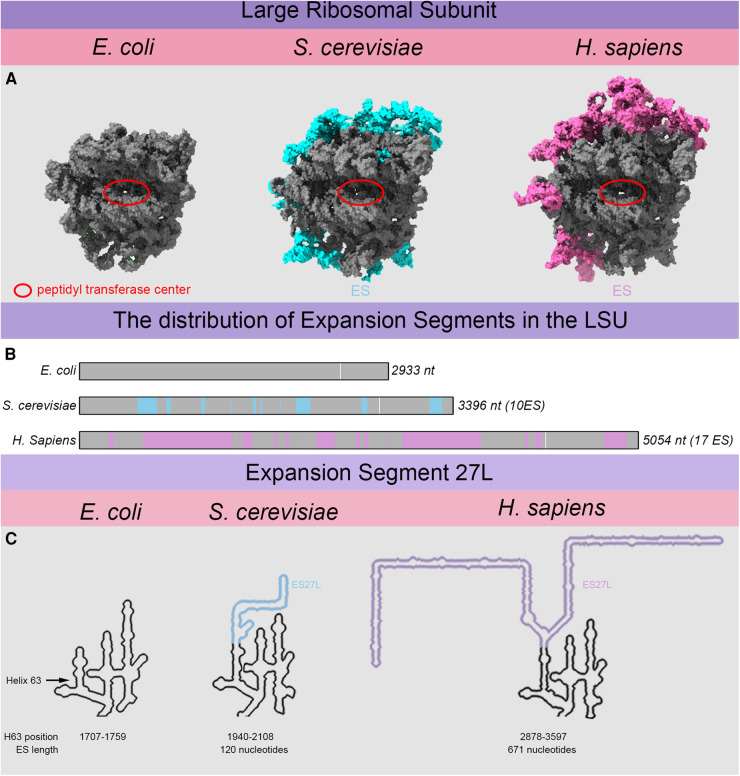
LSU rRNA expansion and the evolutionary growth of helix 63 into expansion segment 27L. (**A**) *E. coli*, *S. cerevisiae* and *H. sapiens* LSU rRNA structures were aligned and displayed to proportion based on structures (8cgv, 4v88 and 5aj0) to illustrate the expansion. The expansions are highlighted in cyan for yeast or hot pink for human ribosomes. The extent to which expansion occurred is underestimated in these figures since many of the large ES cannot be resolved. ES27L is one of the segments displaying high flexibility and which can only be visualized upon capture with interaction partners. (**B**) The rRNA backbone found in the bacterium E. coli is displayed as a bar and yeast cyan) and human (hot pink) expansion segments are indicated. White bars show the location of the peptidyl-transferase center. (**C**) The expansion of bacterial helix 63 into ES27L is illustrated based on secondary structures from RNA central [5]. The arrow indicates the position of the original helix 63 in *E. coli* that expanded in *S. cerevisiae* and *H. sapiens*. The colored part of the helix indicates the specific expansion of helix 63 in yeast (cyan) and human (hot pink).

**Figure 2.**
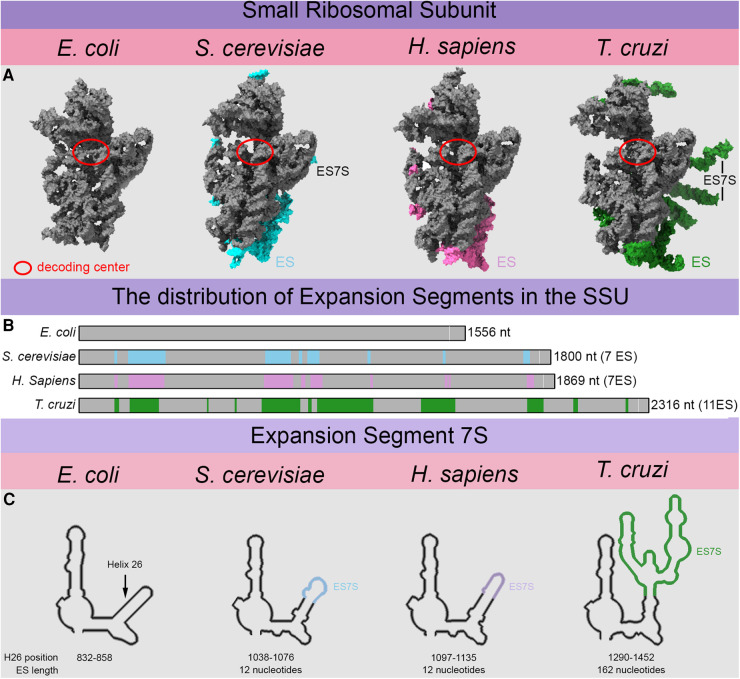
SSU rRNA expansion and the evolutionary growth of helix 26 into expansion segment 7S. (**A**) *E. coli*, *S. cerevisiae*, *H. sapiens* and *T. cruzi* SSU rRNA structures were aligned and displayed to proportion based on structures (8cgv, 4v88, 5aj0 and 7ase) to illustrate the rRNA expansion. The expansions are highlighted in cyan for yeast, hot pink for human and green for trypanosomal ribosomes. The extent to which expansion occurred is underestimated in these figures since many of the large ES cannot be resolved. (**B**) The bars illustrate the bacterial SSU rRNA backbone with yeast (cyan), human (hot pink) and Trypanosoma-specific (green) expansions. White bars indicate the position of the decoding residues A1494 and A1495 in *E. coli* or their conserved counterparts in the other species. The absolute length is given for each species. (**C**) Illustration of the expansion of ES7S from bacterial helix 26 based on secondary structures from RNA central [5]. The arrow indicates the position of the original helix 26 in *E. coli* that expanded in *S. cerevisiae*, *H. sapiens* and especially in *T. cruzi*.

Of special interest is the immense expansion of the rRNA observed (Figure 1) when comparing bacterial, yeast and mammalian ribosomes [4]. In the case of the mammalian (*Homo sapiens*) 28S rRNA, the structural backbone of the LSU, is more than 2000 nt longer than its *Escherichia coli* counterpart, the 23S rRNA. When comparing *E. coli* 23S and *Saccharomyces cerevisiae* 25S rRNA, the expansion is modest reaching 490 nt. These nucleotides are not randomly inserted but cluster in helices at conserved positions, so-called rRNA expansion segments (ESs) [4,6,7]. Their location at the periphery ensures the integrity of the functionally crucial centers figure 1 and figure 2. The yeast 25S rRNA harbors 14 expanded regions with different degrees of complexity ranging from short helix extensions (e.g. ES41L) to insertions of complex segments harboring multiple helices, such as ES27L [6]. To illustrate, ES27L extends from the 44 nt long helix 63 within the *E. coli* ribosome (Figure 1). In yeast, the same helix is expanded by ∼120 nt and contains a slight bifurcation while in humans, expansion progressed to a staggering ∼670 nt featuring two large, flexible helices (Figure 1C). This single expansion accounts for almost one third of the overall expansion observed within the human 28S rRNA [4].

In *S. cerevisiae*, the SSU, which displays less dramatic rRNA expansion, harbors five ESs spanning a wide range of sophistication as well [6]. The rRNA constituting the SSU serves as a fine example of divergent evolution in different evolutionary clades. *S. cerevisiae* and *H. sapiens*, together with most other relevant model organisms belong to the clade of ophistoconts. Their 18S rRNAs display only minor structural differences possibly due to their relatively close relation (Figure 2). When studying evolutionary distant clades such as discobids, major differences within the 18S rRNA can be observed. Discobids such as *Trypanosoma brucei* or *Trypanosoma cruzi* show immense expansion of the small 18S rRNA reaching 2319 nt in length as compared with the yeast 18S rRNA composed of 1798 nt. Here, the ES emerging from conserved positions show much greater expansion than their opisthokonts counterparts [8,9]*.* Clade-specific expansion is beautifully illustrated by ES7S (Figure 2), a short expansion of bacterial helix 26 by only 12 nt in yeast and humans [10]. In *T. cruzi* however, that same helix 26 expanded to a length of ∼160 nt forming two helices at the platform of the SSU [8].

The need to transcribe much larger rRNAs comes at immense costs for the cell; thus, it is conceivable that ES offer an evolutionary advantage. To date, the accepted hypothesis is that the expansions allowed additional options for regulation in a protein biosynthesis process that has become more complex in eukarya as compared to bacteria and archaea. How exactly an ES-driven regulation might work, is barely understood and only now, the first concepts are emerging. This review aims to highlight the studied roles of two chosen ESs (ES27L and ES7S), to infer the consequences of their absence and to address how evolution took different roads in evolutionarily distant organisms.

## ES 27L and its role in factor recruitment to the peptide exit tunnel

**Figure 3.**
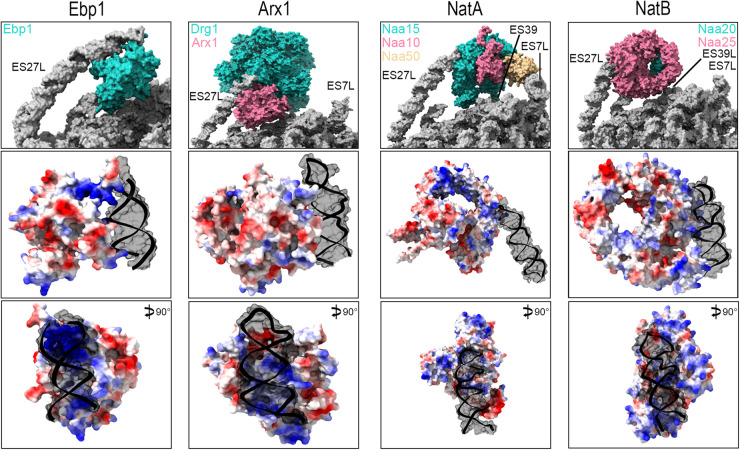
ES27L recruits peptide channel interactors cotranslationally and during ribosome biogenesis. ES27L recruits Ebp1 (first column, pdb: 6sxo), Arx1 (second column, pdb: 7z34), NatA (third column, pdb: 6hd7) and NatB (fourth column, pdb: 8bip). The top row displays the ES27L (grey) interaction with various protein partners (colored). ES7L as part of the interaction surface is indicated. The second and third row displays the charge distributions of protein partners at the ES27L binding site. Structures are rotated 180° vertically and 90° horizontally with respect to the first row. The ribbon and grey surface indicate ES27L, the red surface indicates negative charge and the blue surface indicates positive charge. The third row is rotated vertically by 90° with respect to row 2. Figures were prepared using ChimeraX software.

rRNA ESs were described in most cases to be embedded into a layer of eukaryote-specific ribosomal proteins [6]. While this notion is true, the large ESs ES7L, ES27L and ES6S often elude cryo-electron microscopic capture suggesting their flexibility [7]. In yeast, ES7L_b_, the longest expanded helix, is firmly embedded into the ribosome structure facing from the back of the LSU toward the ribosomal P-Stalk [6]. ES7L_a_, however, is shorter, yet very flexible rendering it a potentially newly evolved factor recruitment platform (Figure 3) [6]. ES27L, located at the back of the LSU, appears to be unique in that it occupies two specific states in cryo-EM structures (coined 27L_in_ and 27L_out_) [7]. In the 27L_in_ conformation, the RNA faces toward the E-site tRNA exit and the L-Stalk, whereas in the 27L_out_ conformation, it is located toward the nascent peptide exit tunnel (PET) [7]. In the 27L_out_ conformation, recent evidence has shown, that it is part of a very dynamic recruitment platform that binds peptide tunnel interacting proteins. Associated proteins function in ribosome biogenesis and nascent peptide quality assurance; key cellular processes, that highlight the importance of this ES in eukaryotic cells. In the following paragraph, we want to compile recent findings on which proteins are directly recruited to the translation machinery via ES27L, and what their functional implications might be.

### Ebp1 and methionine amino peptidase

The metazoan-specific ErbB3 receptor-binding protein (Ebp1) is an abundant ribosome-associated protein both in HeLa [11] and in mouse embryonic neuronal stem cells with implications for cancer and brain development [12,13]. While early studies identified Ebp1 to bind the ErbB3 receptor, more recent studies suggest substantial binding to the PET, an interaction mediated by ES27L (Figure 3) [11,14]. Ebp1, belonging to the methionine amino peptidase (MetAP) protein family, has two contact points with ES27L, that are evolutionarily conserved on the rRNA and (partly) protein level. The first contact point contains common MetAP fold regions of the protein whereas the second is Ebp1-specific and located in its C-terminus (Figure 3) [11]. This binding mode locates an electronegative concavity directly over the PET vestibule, the cytosol-exposed part of the PET, thus shielding the nascent chain. The interaction is maintained during early translation until ∼80 amino acids are synthesized when Ebp1 is displaced. Earlier displacement occurs in the case of signal peptides after ∼40 amino acids [13]. At this point, the signal recognition particle occupies the peptide tunnel vestibule to guide the ER recruitment of the translating ribosome. Intriguingly, depletion of Ebp1 results in stalled ribosomes at the start codon, suggesting that Ebp1 is required for translation of the first mRNA codons or during the release of initiation factors before elongation starts [13]. As there is no data on ES27L ablation in mammalian systems, the field is blind to the contribution of ES27L to Ebp1 binding at the ribosome.

The structurally conserved protein MetAP displays a very similar binding mode as compared with Ebp1. It is also guided by ES27L and covers the peptide tunnel exit with its electronegative nascent chain binding pocket [13,15]. In contrast with Ebp1, however, it is enzymatically active and removes the initial methionine of its nascent chain substrates.

### ARX1

The MetAP fold is also present in a protein involved in ribosome biogenesis, Arx1. ES27L binds in a very similar fashion as compared with Ebp1; one binding site is MetAP fold-specific and the second is in Arx1-specific extensions of the fold (Figure 3) [16]. Here, the ES27L-Arx1 interface is large, likely substantially contributing to the binding affinity. As observed for Ebp1 and MetAP, the PET is shielded by Arx1 (Figure 3), however, the binding pocket does not display the observed electronegativity [13]. In the immature 60S particle, no positively charged nascent chain N-terminus must be shielded, which likely explains a different charge distribution in Arx1. During cytosolic LSU maturation, Arx1 appears to play the role of an extended recruitment platform. First, it recruits the AAA-ATPase Drg1 (Figure 3), which is further stabilized by interactions with ES27L [16]. Drg1, a molecular ratchet, then extracts Rlp24 from the nascent ribosome, a crucial step during the cytosolic phase of maturation.

Once Drg1 has extracted Rlp24, it dissociates and the Arx1 interaction partner changes. Rei1 binds to probe the PET for accurate assembly, which will allow nascent chain passage during translation. To that end, it inserts its C-terminus into the peptide tunnel, mimicking a nascent chain [16]. If that step is unsuccessful, the C-terminal domain remains located in the cytosol and blocks further maturation near the PET. During that step, Alb1 (another maturation factor) is already bound to the 60S precursor, directly interacting with Arx1. The role of that interaction is not fully understood yet, but it seems to regulate Arx1 dynamics. The Arx1 binding comes to an end when Rei1 has successfully inserted its C-terminus [16]. The Arx1-Rei1 interface then forms the binding site of the Hsp40 Jjj1, which in turn recruits the Hsp70 Ssa1/2 [17,18]. The chaperone activity likely extracts the Rei1 C-terminus from the tunnel and destabilizes the complex. Eventually, Arx1, Rei1, and Alb1 dissociate, which represents the next major milestone during cytosolic 60S maturation [17,19].

While the specific contribution of ES27L to ribosome biogenesis factor recruitment has not been addressed, several lines of evidence indicate that the absence of ES27L in yeast causes biogenesis defects. Reduced levels of free 60S subunits have been observed in ES27L deletion strains [20,21]. Moreover, accumulations of ‘halfmers’ can be observed in the yeast ES27L ablation strain [21]. Halfmers indicate the presence of a 48S complex waiting for a 60S subunit to join and are considered signs of a 60S shortage [22].

### N-terminal acetyltransferase complexes A and B

A set of three studies conducted in baker's yeast highlighted the role of ES27L in the recruitment of the N-terminal acetyltransferase (Nat) complex NatA [21,23,24]. Engineered yeast cells that exclusively express ES27L_b_-ablated ribosomes are viable and fit under normal conditions. Ribosomes, however, fail to recruit the N-terminal acetylation complexes NatA and NatB [21]. NatA acetylates A-, S-, T-, V-, C- and partly G-starting N-termini after MetAP mediated cleavage of methionine [25]. NatB acts on methionine-retained nascent chains starting with MD-, ME-, MQ- and MN. NatB moreover, has not yet been reported to act posttranslationally, whereas NatA has posttranslational activity [25]. Reduced levels of NatA/B at the translating ribosome resulted in markedly reduced acetylation of a subset of NatA substrates accompanied by increased proteomic aggregation tendency [21]. The exposed charge in proteins lacking acetylation has the potential to alter their folding also affecting their interactions with auxiliary factors [26]. The loss of protein acetylation in ES27L_b_ ablated cells can moreover alter the cellular half-life of proteins via enhanced or decreased proteasomal degradation. Depending on the amino acid context, acetylations can form a degron tag or mask it thus modulating the cellular half-life of proteins [26–29]. By recruiting NatA/B to the ribosomes and therefore ensuring the efficient N-terminal acetylation of nascent peptide chains ES27L_b_ plays a crucial role in proteome integrity.

As compared with Ebp1 or MetAP, NatA (Naa10, harboring the active site) and NatB (Naa20) do not directly occupy the peptide tunnel vestibule but are located roughly 50 and 65 Å from it respectively (Figure 3) [15,23]. This distance allows MetAP to bind to the PET directly, thus acting before NatA/B, which is essential. While simultaneous binding has been postulated, the binding of ES27L to each of the proteins is mutually exclusive [15]. This raises the conundrum of whether indeed simultaneous binding can occur or whether ES27L prefers binding one factor over the other.

In terms of their binding mode to ES27L, NatA and NatB differ from one another. In both cases, the binding site is composed of a positively charged surface to interact with the RNAs phosphate backbone (Figure 3). NatA, however, is clamped between ES27L_b_ and ES7L_a_ with further contacts to ES39L [23], while NatB contacts only ES27L_b_ [15]. Moreover, NatA is contacted exclusively by the tip of ES27L_b_, whereas NatB has a much larger interaction surface (Figure 3). This different binding modality results in a much higher dependence of NatB-association on ES27L_b_ as has been observed in ES27L_b_ ablated ribosomes [21]. To date, it remains uncertain, which part of the translatome requires cotranslational N-terminal modification and which part can be modified posttranslational. It appears however that NatB targets rely more heavily on cotranslational modification, therefore making them a likely subgroup of aggregation-prone proteins upon ES27L ablation.

### A constant fight or a dance? How is factor binding coordinated?

The number of known translation cofactors binding in the vicinity of the PET is large and will likely increase with more research. Many different nascent chain shielding, processing and membrane targeting steps are guided by these factors and require their ribosome association and therefore displacement of other factors. How is that choreography maintained? Only limited answers exist so far. Factors catalyzing the modification of the N-terminus apparently can bind simultaneously [15,23] and must act early during translation. Later acting factors might then take over. In that scenario, ES27L might play a crucial role; its conformational dynamics allow for altered binding affinity of factors and can therefore contribute to a handing-over mechanism. The degree of ES27L stabilization in the tunnel conformation might define a time window for factor recruitment. The ES27L interaction surfaces of MetAP folds (found in EBP1 or MetAP) are very similar to each other but could still allow for fine-tuned regulation [13]. The NatA/B interaction also differs and thus interaction capacities might be different as observed in ES27L ablation strains. Further research will need to elucidate that choreography.

## ES7S plays diverse roles in different evolutionary clades

### ES 7S in discobids

The far distant clade of discobids contains the kinetoplastids, Trypanosoma, known parasites causing diseases in humans and cattle. These organisms display a fascinating and unique ribosome biology. Not only is their 25S ribosomal RNA fragmented but both, 25S and 18S rRNA display clade-specific features of ESs (Figure 2) [8,9].

Prominent among them is ES7S, a large extension of bacterial helix 26, which displays a bifurcated structure facing toward the cytosol (Figure 2). With its two arms, it effectively engages initiation factor 3 during translation initiation [8]. Here it embraces the core component of eIF3, eIF3c. In addition, there are contacts to eIF3a both of which enable efficient loading of eIF3 to the 40S subunit. This large binding surface is composed of rRNA which required a redistribution of charges within eIF3c, as compared with mammalian eIF3c. This charge redistribution has been observed, suggesting the important contribution of ES7S to eIF3 binding during initiation [8]. Once eIF3 dissociates, ES7S dramatically changes its conformation, thus avoiding eIF3 binding during elongation. The clamp formed by ES7S is opened by rotation of helix ES7S_b_ [8,9]. The structural rearrangements are observed in other large ES such as ES27L in ophistokonts [7] and appear to be part of the regulatory strategy. Deletion studies elaborating on the effects of ES7S expansion in discobids are missing to date, thus it remains uncertain whether eIF3 binding is highly reliant on ES7S expansion in trypanosomes.

### ES 7S in ophistokonts

In ophistoconts, including established model organisms such as *S. cerevisiae* and *H. sapiens*, ES7S only marginally expanded from bacterial helix 26. In both cases, the helix grew by solely 12 nt (Figure 2). This slight increase did not allow for bifurcation and provided ES7S with a reduced solvent-exposed surface as compared with *T. cruzi* ribosomes [8]. Consequently, its ability to recruit factors as seen in Trypanosomes is limited. A recent study has found ES7S to be involved in regulating translation elongation at the step of translocation [10]. For the first time, the data provides evidence for an ES as a direct regulator of translation velocity and dynamics.

Rauscher et al*.* [10] ablated the ES7S in yeast leaving behind a helix resembling the bacterial helix 26 and observed reduced cell proliferation coupled to reduced and error-prone translation. Rare codons were especially vulnerable to amino acid misincorporations leading to their apparently faster translation. Cotranslationally folding proteins suffered from the deregulated translational speed as indicated by decreased steady-state levels and stability at the proteomic scale. Intriguingly, when the yeast ES7S sequence was exchanged for that of human rRNA, an increased resistance towards miscoding-inducing drugs was observed, suggesting that the sequence of the ES can modulate the accuracy of translation. Last, *in vivo* structural probing studies in *S. cerevisiae* harboring ablated ES7S revealed an altered population of translation elongation intermediate states which was linked to a defect in progression from a late PRE-translocation state to a POST state ribosome. This study identified for the first time a role of ESs other than recruitment of factors and suggests that evolutionary pressure, to achieve higher translation accuracy, could be met by slightly modulating one rRNA helix within the SSU [10].

## Perspectives

- Translation regulation is still far from fully understood and the contribution of ribosomal features has barely been investigated. They however do play a role in ribosome biology and consequently in proteomic integrity.
- Initially, ESs were identified as recruitment platforms for initiation and biogenesis factors but also for cotranslationally acting enzymes. Recently, evidence mounted that ESs display conformational dynamics that enable them to modulate translation elongation dynamics.
- Many ESs have not been studied yet, therefore their full potential in factor recruitment, maturation and translation dynamics remains to be determined. It appears that rRNA expansions contribute to protein biosynthesis by adding a variety of different, mostly regulative, roles to eukaryotic ribosomes, thus suggesting an underlying evolutionary pressure for their emergence.

## Abbreviations

ES: expansion segment
LSU: large subunit
MetAP: methionine amino peptidase
PET: peptide exit tunnel
PTC: peptidyl transferase center
SSU: small subunit
